# Supplementary material for: Identification and functional analysis of PIN family genes in Gossypium barbadense
Source: PeerJ. 2022 Oct 18;10:e14236. doi: 10.7717/peerj.14236 (PMC9586078; doi:10.7717/peerj.14236)
Supplement: Supplemental Information 1 [file peerj-10-14236-s001.pdf]

Plant Indole-3-acetic acid；Auxin

FOR RESEARCH USE ONLY

Drug Names

Generic Name：**Plant Indole-3-acetic acid；Auxin (IAA；Auxin) ELISA Kit.**

Purpose

This kit allows for the determination of IAA；Auxin concentrations in Plant tissue homogenates and other biological fluids.

Principle of the assay

The kit assay Plant IAA；Auxin level in the sample, use Purified Plant IAA；Auxin antibody to coat microtiter plate wells, make solid-phase antibody, then add IAA；Auxin to the wells, Combined antibody which With HRP labeled, become antibody-antigen-enzyme-antibody complex, after washing Completely, Add TMB substrate solution,TMB substrate becomes blue color At HRP enzyme-catalyzed, reaction is terminated by the addition of a sulphuric acid solution and the color change is measured spectrophotometrically at a wavelength of 450 nm. The concentration of IAA；Auxin in the samples is then determined by comparing the O.D. of the samples to the standard curve.

Materials provided with the kit

Materials provided with the kit	48determinations	96 determinations	Storage
User manual	1	1	
Closure plate membrane	2	2	
Sealed bags	1	1	
Microelisa stripplate	1	1	2-8℃
Standard	0.3ml×6 bottle	0.3ml×6 bottle	2-8℃
HRP-Conjugate reagent	5ml×1 bottle	10ml×1 bottle	2-8℃
Sample diluent	3ml×1 bottle	6ml×1 bottle	2-8℃
Chromogen Solution A	3ml×1 bottle	6ml×1 bottle	2-8℃
Chromogen Solution B	3ml×1 bottle	6ml×1 bottle	2-8℃
Stop Solution	3ml×1 bottle	6ml×1 bottle	2-8℃
20×Wash solution	15ml×1 bottle	25ml×1 bottle	2-8℃

Note: Standard concentration was followed by:
20、10、5、2.5、1.25、0 μmol/L.

Specimen requirements

1. **serum-** coagulation at room temperature 10-20 mins，centrifugation 20-min at the speed of 2000-3000 r.p.m. remove supernatant, If precipitation appeared, Centrifugal again.
2. **plasma-**use suited EDTA or citrate plasma as an anticoagulant,mix 10-20 mins ,centrifugation 20-min at the speed of 2000-3000 r.p.m. remove supernatant, If precipitation appeared, Centrifugal again.
3. **Urine**-collect sue a sterile container, centrifugation 20-min at the speed of 2000-3000 r.p.m. remove supernatant, If precipitation appeared, Centrifugal again. The Operation of Hydrothorax and cerebrospinal fluid Reference to it.
4. **cell culture supernatant**-detect secretory components, collect sue a sterile container, centrifugation 20-min at the speed of 2000-3000 r.p.m. remove supernatant,detect the composition of cells, Dilut cell suspension

with PBS（PH7.2-7.4）, Cell concentration reached 1 million / ml, repeated freeze-thaw cycles, damage cells and release of intracellular components, centrifugation 20-min at the speed of 2000-3000 r.p.m. remove supernatant, If precipitation appeared, Centrifugal again.

5. **Tissue samples**- After cutting samples, check the weight,add PBS（PH7.2-7.4）, Rapidly frozen with liquid nitrogen, maintain samples at 2-8℃ after melting,add PBS（PH7.4）, Homogenized by hand or Grinders, centrifugation 20-min at the speed of 2000-3000 r.p.m. remove supernatant.
6. extract as soon as possible after Specimen collection,and according to the relevant literature, and should be experiment as soon as possible after the extraction. If it can't, specimen can be kept in -20 ℃ to preserve, Avoid repeated freeze-thaw cycles.
7. Can't detect the sample which contain NaN3, because NaN3 inhibits HRP active.

Assay procedure

1. Add standard: Set Standard wells, testing sample wells. Add standard 50μl to standard well.

2.add sample：Set blank wells separately (blank comparison wells don't add sample and HRP-Conjugate reagent, other each step operation is same). testing sample well. add Sample dilution 40μl to testing sample well, then add testing sample 10μl (sample final dilution is 5-fold), add sample to wells , don't touch the well wall as far as possible, and Gently mix.

3.add enzyme：Add HRP-Conjugate reagent 100μl to each well, except blank well.

4.Incubate: After closing plate with Closure plate membrane ,incubate for 60 min at 37℃.

5.Configurate liquid: 20-fold wash solution diluted 20-fold with distilled water

and reserve.

6.washing：Uncover Closure plate membrane, discard Liquid, dry by swing, add washing buffer to every well, still for 30s then drain, repeat 5 times, dry by pat.

7.color：Add Chromogen Solution A 50ul and Chromogen Solution B to each well, evade the light preservation for 15 min at 37℃

8.Stop the reaction：Add Stop Solution 50μl to each well, Stop the reaction(the blue color change to yellow color).

9.assay：take blank well as zero , Read absorbance at 450nm after Adding Stop Solution and within 15min.

Important notes

1. The kit takes out from the refrigeration environment should be balanced 15-30 minutes in the room temperature, ELISA plates coated if has not use up after opened, the plate should be stored in Sealed bag.
2. washing buffer will Crystallization separation, it can be heated the water helps dissolve when dilute . Washing does not affect the result.
3. add Sample with sampler Each step, And proofread its accuracy frequently, avoids the experimental error. add sample within 5 mins, if the number of sample is much , recommend to use Volley .
4. if the testing material content is excessively higher (The sample OD is bigger than the first standard well),please dilute Sample (n-fold), Please diluente and multiplied by the dilution factor.（×n×5）.
5. Closure plate membrane only limits the disposable use, to avoid cross-contamination.
6. The substrate evade the light preservation.
7. Please according to use instruction strictly, The test result determination must take the microtiter plate reader as a standard.
8. All samples, washing buffer and each kind of reject should according to infective material process.
9. Do not mix reagents with those from other lots.

Calculate

Take the standard density as the horizontal, the OD value for the vertical ,draw the standard curve on graph paper, Find out the corresponding density according to the sample OD value by the Sample curve, multiplied by the dilution multiple, or calculate the straight line regression equation of the standard curve with the standard density and the OD value ,with the sample OD value in the equation, calculate the sample density,

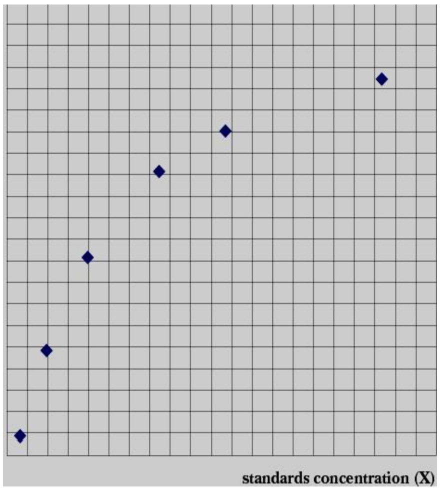

This chartis for reference only

Assay range

0.625 μmol/L - 20 μmol/L

Sensitivity

The minimum detectable dose is typically less than 0.1 μmol/L

Storage and validity

1．Storage： 2-8℃.

2．validity： six months.
